# 554. Association of *emr* and *mdt* Genes in Carbapenem Resistant Acinetobacter *baumannii* with Clinical Outcome in A Tertiary Care Hospital

**DOI:** 10.1093/ofid/ofad500.623

**Published:** 2023-11-27

**Authors:** M O N I K A CHAUDHARY, Deepak Kumar, Gopal Krishana Bohra, Naresh Kumar Midha, Durga Shankar Meena, Vibhor Tak, M K Garg, Sadik Mohammed, Amit Kumar Rohilla

**Affiliations:** AIIMS, JODHPUR, JODHPUR, Rajasthan, India; All India Institute of Medical Sciences, Jodhpur (India), Jodhpur, Rajasthan, India; AIIMS Jodhpur, Jodhpur, Rajasthan, India; AIIMS, Jodhpur, Rajasthan, India; AIIMS, Jodhpur, Rajasthan, India; All India Institute of Medical Sciences, Jodhpur, Jodhpur, Rajasthan, India; AIIMS, Jodhpur, Rajasthan, India; ALL INDIA INSTITUTE OF MEDICAL SCIENCES, JODHPUR, jodhpur, Rajasthan, India; ALL INDIA INSTITUTE OF MEDICAL SCIENCES, JODHPUR, jodhpur, Rajasthan, India

## Abstract

**Background:**

Carbapenem Resistant Acinetobacter *baumannii* (CRAB) is one of the most common cause of nosocomial infections in ICUs and associated with high mortality. Because the organism can acquire resistance to multiple drugs by various mechanisms, very few treatment options have been left. We evaluated the genes which conferred multidrug resistance to clinically virulent CRAB isolated from 4 critically ill patients and correlated them with their clinical outcome.

**Methods:**

It was a small prospective study under the Antimicrobial Stewardship Program in a tertiary care hospital of Western Rajasthan, India. It included 4 critically ill patients who were diagnosed with hospital acquired CRAB bactaeremia in the month of October and November 2022. They were admitted in ICU and were on ventilator > 48 hours post admission with no comorbidities. Microbiologically proven CRAB isolated from the blood of these 4 patients were sent for Whole Genome Sequencing(WGS).These samples were sequenced using Novaseq with a read length of 151 bp. 2 different strains of Acinetobacter *baumannii* were identified in these 4 samples- XH731 and WKA02. Denovo assembled fasta sequence was annotated using Prokka to predict CDS, genes and other features of genome.

**Results:**

Table1 is showing the presence of *emr* and *mdt* genes in the CRAB strains isolated from the blood of 4 patients. They are responsible for colistin resistance and multidrug resistance by formation of efflux pumps respectively. CRAB isolated from patient C was having *emrA1, emrA2, emrA3, emrA4, emrA5, mdtE, mdtK, mdtA1, mdtA2, mdtA3, mdtA4, mdtC*. This patient succumbed to death within 5 days of admission despite of appropriate combination therapy, implying very high virulence of CRAB harboring these genes. Moreover, in patient D extra number of drug resistant genes were found as compared to patient A and B.

Multidrug resistant genes found in CRAB isolated from 4 patients of AICU and their outcome

**Figure:**
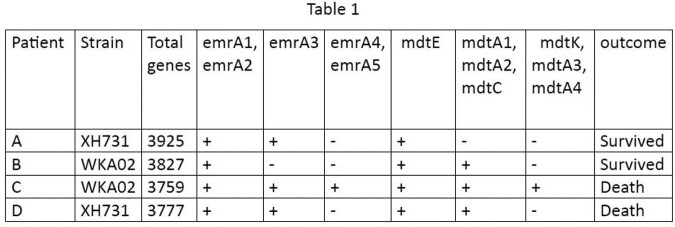

**Conclusion:**

Increased virulence of CRAB due to its resistance to almost every drug has made its management very difficult. WGS can be used to identify certain genes which may be responsible for rapid progression of disease. Presence of *emrA4, emrA5, mdtK, mdtA3* and *mdtA4* genes responsible for formation of efflux proteins may be associated with increased mortality as observed in our study.

**Disclosures:**

**All Authors**: No reported disclosures

